# An Analysis of Foot and Ankle Device Recalls by the Food and Drug Administration

**DOI:** 10.7759/cureus.3123

**Published:** 2018-08-09

**Authors:** Carl Pellerin, Vinod Panchbhavi, Cory F Janney

**Affiliations:** 1 Department of Orthopaedic Surgery and Rehabilitation, University of Texas Medical, Galveston, USA; 2 Department of Orthopedics Surgery and Rehabilitation, University of Texas Medical Branch, Galveston , USA; 3 Department of Orthopedics, Naval Medical Center, San Diego, USA

**Keywords:** foot, ankle, fda, device, recall

## Abstract

Introduction

Orthopaedic devices represent 12% of all medical device recalls. Products are approved through pre-market approval (PMA) or the 510(k) premarket notification process. No previous evaluation was found in the literature evaluating foot and ankle device recalls. The field of foot and ankle subspecialty has seen a rapid growth in innovation related to implants in recent years.

Methods

The Food and Drug Administration (FDA) Device Recall database was evaluated for all foot and ankle devices from 2007 through 2017 for the manufacturer, process of approval, type of implant, recall class, dates of initiation and termination of the recall, manufacturer determined reason, quantity affected, and distribution within the United States or internationally.

Results

A total of 161 products from 33 companies were identified with 158 (98.1%) approved through the 510(k) process. The most common reason for device recall was due to the device breaking intraoperatively or postoperatively. The average length of the recall was 487.5 days.

Conclusions

Device recall is not an uncommon event with the majority of products approved through the less demanding 510(k) process.

## Introduction

Medical device recalls are common. According to the U.S. Food and Drug Administration (FDA), there were 20,093 recalls instituted by 1,641 manufacturers between November 2002 and December 2012 [1]. The incidence of recalls has not decreased over time. This unfortunate circumstance was confirmed as the FDA implemented 13,937 additional device recalls from 2013 through 2017. Of these additional product recalls, 3,202 occurred in 2017 alone [2].

The FDA is responsible for overseeing and regulating medical devices within the United States. Medical devices are typically approved through two methods: the premarket approval (PMA) process and the 510(k) premarket notification process. The PMA process has proven to be more expensive and time-consuming as it requires clinical evidence for authorization [3]. On the other hand, the 510(k) premarket notification is an accelerated process that omits medical devices from the clinical trial requirements as long as the device is “substantially equivalent” to an alternatively utilized medical device [4]. Defective medical devices have been recalled after gaining prior FDA authorization regardless of approval process utilized.

The United States medical device market has attracted a large sum of wealth and expenses throughout the years. In fact, this market was estimated to be over $133 billion in 2016 [5]. The economic impact for the numerous categories of medical devices varies significantly [6]. In the field of orthopaedics, the majority of medical devices are utilized for procedures such as joint replacement (projected U.S. market of $10.3 billion in 2018) and fracture management (projected U.S. market of $4.3 billion in 2015) [3, 7-8].

Although various medical devices have been utilized in operating rooms throughout the years, there is limited research evaluating the rationalization behind orthopaedic device recalls. Orthopaedic devices represent 12% of all medical device recalls [9]. Yet, no previous study was found in the literature examining foot and ankle devices with regards to recalls. Thus, this paper aims to compile and evaluate the reasons behind why ankle and foot orthopaedic devices have been recalled between 1 January 2007 through 31 December 2017. We hypothesized that products approved through the 510(k) approval process would have a higher recall rate.

## Materials and methods

The Food and Drug Administration Device Recall database was queried for all foot and ankle devices from 1 January 2007 through 31 December 2017 by searching for the following terms: foot, ankle, toe, tibiotalar, subtalar, talus, calcaneus, navicular, cuboid, cuneiform, metatarsal, and phalanx [10]. Duplicates were removed from the search. The products were then verified as foot and ankle devices which may be used by a foot and ankle orthopaedic surgeon or podiatrist. Each recall was then used to evaluate the manufacturer, type of implant, recall class, date of initiation of the recall, date of termination of the recall, manufacturer determined reason, FDA determined reason, quantity affected, submission type, and the distribution within the United States or internationally.

## Results

**Figure 1:**
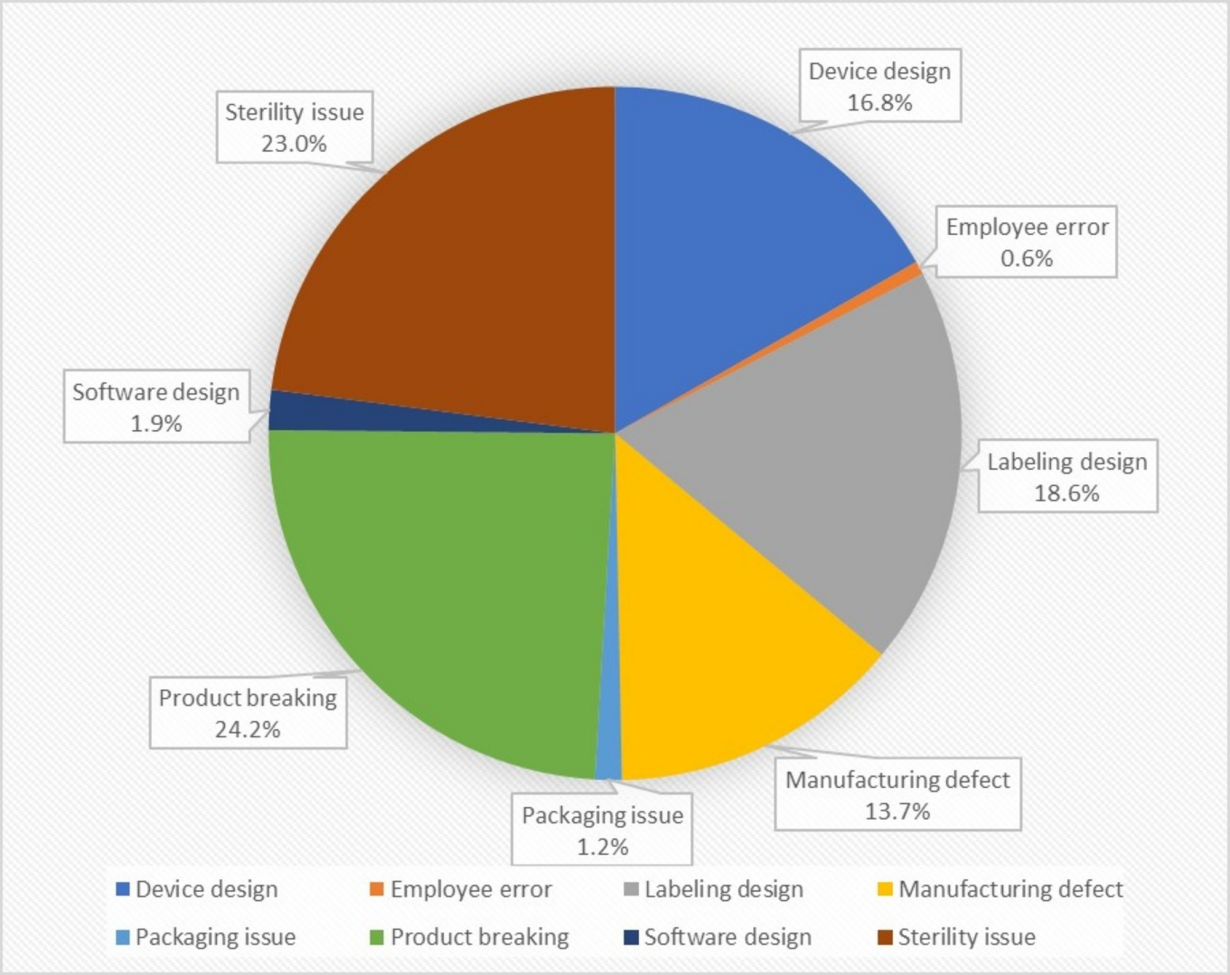
Manufacturer-determined reasoning for all products recalled.

A total of 161 products from 33 different companies were identified (Table 1). The most common reason products were recalled was due to the device breaking intraoperatively or postoperatively, with one company recalling 78,594 suture anchors due to fractures of the hardware requiring revision surgery. Device breakage accounted for 39 of the 161 medical device recalls of implants or devices used during surgery with 12 occurring interoperatively and 26 postoperatively. One product was a prosthetic foot that could break under normal usage causing compromise to stability and function. The second-most common reason for recall was sterility concerns, which accounted for 37 recalls. Labeling issues and device design issues were also common reasons for recall, accounting for 30 and 27 recalls respectively. Employee error caused the least amount of recalls, with only one product removed from the market (Figure 1).

**Table 1:** Manufacturer-determined reasoning for recall of the devices from January 1, 2007 through December 31, 2017.

Recall reason	Number of products recalled
Product breakage	39
Sterility issue	37
Labeling design	30
Device design	27
Manufacturing defect	22
Software design	3
Packaging issue	2
Employee error	1
Total products	161

The average length of the active recall time from initiation to termination was 487.5 days. The smallest number of recalled products in commerce were two sizes of Mitek SpiraLok anchors (DePuy Mitek Inc., Raynham, Massachusetts, US) with zero distributed that were recalled secondary to concerns for hardware failure. The largest number of recalled products were Synthes screws (Synthes Products LLC, West Chester, Pennsylvania) (cancellous, cortical, and cancellous of various sizes) with 19,497,844 screws recalled due to labeling issues.

**Figure 2:**
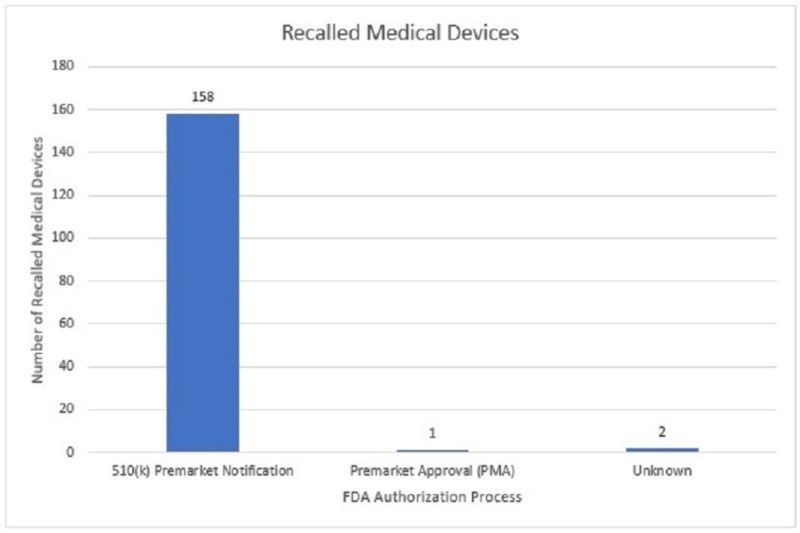
FDA approval process utilized by the foot and ankle devices recalled during the selected timeframe. FDA - Food and Drug Administration.

Of the 161 medical devices that were recalled, 158 (98.1%) were approved through the 510(k) premarket notification process. Conversely, only one product was authorized through the premarket approval (PMA) process. The submission procedure was not indicated for the remaining two medical devices (Figure 2).

## Discussion

During the recall process, it is the manufacturers’ duty to notify surgeons. The surgeons should then identify the patients affected and notify them, making sure they understand the reasons for the recall as well as what the risks are to the patient [11]. Although the surgeon may be concerned about litigation, the cases are categorized as product liability cases and generally are not considered the surgeon’s fault [12]. These patients should be followed frequently but prophylactic surgery is generally not recommended [13].

The overwhelming majority of recalls involved products approved through the 510(k) premarket notification process. This process does not involve the same stringent standards as the PMA application process. The PMA process requires clinical evidence before approval is granted, often requiring evidence on the level of randomized clinical trials or prospective data compared with historical controls [3-4, 14]. This study further supports the idea that products approved through the PMA process are less likely to be recalled [1, 15]. There is a lack of clinical data with products approved through the 510(k) process that may cause the devices to carry a greater risk to patients. With regards to total hip arthroplasty implants, it is estimated that only 15% of these devices had data on clinical effectiveness published [16].

The 510(k) process allows a company to fast-track approval of a device by claiming it “substantially equivalent” to a device that has already been approved and marketed. Claiming that one device is equivalent to another device may be illogical even when the predicate comes from the same company [17-18]. This process may also lead to devices being approved on the predicate from another device that was itself approved through the 510(k) process, sometimes leading to decades between the original device and subsequent predicate devices without an increase in evidence of the product working [17].

Products breaking and sterility concerns were the most likely reasons for recall. Issues with products breaking intraoperatively or postoperatively may have been determined previously if the products had undergone the more demanding PMA process for approval. Sterility issues are often related to packaging which may not have been determined through a different testing protocol. 

This study does have several limitations. We focused only on implants utilized within foot and ankle orthopaedic surgery by attempting to identify these products within the FDA database by searching for a wide array of terms within the database. If a product did not register one of the keywords we utilized, we may have missed some of the devices that were recalled. We also were unable to identify the approval process for two devices: the SYMBIONIC LEG (Ossur Americas Inc., Foothill Ranch, California, US) and Centurion Sterile Rubber Bands (#84 Rubber Bands Reorder EB84, Centurion Medical Products Corporation, Howell, Michigan, US). The SYMBIONIC LEG is an integrated prosthetic leg for transfemoral and knee disarticulations that was recalled due to a software design error that failed to notify patients of a low battery before shutting down. The Centurion Sterile Rubber Bands were mislabeled as “latex free” erroneously. These rubber bands did, in fact, contain natural latex rubber and were recalled to prevent patients from having an allergic reaction.

Another limitation is that although the FDA-determined reasons were provided in the database, this may not correlate exactly with the manufacturer’s reason for initiating a recall. Therefore, we chose to focus on the manufacturer’s reasoning for initiating the recall. We were also unable to ascertain the costs associated with the initiation of these recalls or the direct effect on patients as this data is not readily available within the database.

## Conclusions

A large proportion of implants used for foot and ankle surgery approved through 501(k) process were recalled when compared to the proportion of implants approved through the more stringent PMA process. Surgeons should stay abreast of recalls amongst the products that they use frequently. Although recalls are not often considered to be the fault of the operating surgeon, the patient’s best interest is served with close follow up and making sure the patients is knowledgeable regarding the issues associated with the products themselves. Streamlining the PMA process or changing the 510(k) process may enable manufacturers to improve upon the safety of their devices.

## References

[1] Day CS, Park DJ, Rozenshteyn FS, Owusu-Sarpong N, Gonzalez A (2016). Analysis of FDA-approved orthopaedic devices and their recalls. J Bone Joint Surg Am.

[2] (2018). Device recalls in 2017: making sense of the numbers. https://www.raps.org/regulatory-focus%E2%84%A2/news-articles/2018/1/device-recalls-in-2017-making-sense-of-the-numbers.

[3] Yang BW, Iorio ML, Day CS (2017). Orthopaedic device approval through the premarket approval process: a financial feasibility analysis for a single center. J Bone Joint Surg Am.

[4] (2018). U.S. Food and Drug Administration: overview of device regulation. http://www.fda.gov/MedicalDevices/DeviceRegulationandGuidance/Overview/.

[5] (2018). Medical technology spotlight: the medical technology industry in the United States. https://www.selectusa.gov/medical-device-industry-united-states.

[6] ORTHOKNOW ORTHOKNOW (2018). ORTHOKNOW: strategic insights into the orthopaedic industry (2012). https://www.orthoworld.com/index.php/fileproc/index/knowentADJADJorthoknowADJADJ2012ADJADJorthoknow1206LXLXLXpdf.

[7] (2018). U.S. markets for fracture fixation products. https://lsintel.com/market-reports-page.php?id=A326.

[8] (2018). Global market for joint reconstruction and replacement to reach $16.2 billion in 2018. https://www.bccresearch.com/pressroom/hlc/global-market-for-joint-reconstruction-and-replacement-to-reach-$16.2-billion-In-2018.

[9] MEDICAL DEVICES (2018). Medical devices: FDA should enhance its oversight of recalls. https://www.gao.gov/new.items/d11468.pdf.

[10] (2018). U.S. Food and Drug Administration: medical device recalls. https://www.accessdata.fda.gov/scripts/cdrh/cfdocs/cfRES/res.cfm.

[11] Racine J (2018). Orthopedic medical devices: ethical questions, implant recalls and responsibility. R I Med J.

[12] Weinberg J (2013). Johnson & Johnson is facing more than 10,000 lawsuits over an artificial hip that has been recalled. Rev Recent Clin Trials.

[13] Pivec R, Meneghini RM, Hozack WJ, Westrich GH, Mont MA (2014). Modular taper junction corrosion and failure: how to approach a recalled total hip arthroplasty implant. J Arthroplasty.

[14] Fargen KM, Frei D, Fiorella D, McDougall CG, Myers PM, Hirsch JA, Mocco J (2013). The FDA approval process for medical devices: an inherently flawed system or a valuable pathway for innovation?. J Neurointerv Surg.

[15] Zuckerman DM, Brown P, Nissen SE (2011). Medical device recalls and the FDA approval process. Arch Intern Med.

[16] Mahomed NN, Syed K, Sledge CB, Brennan TA, Liang MH (2008). Improving the postmarket surveillance of total joint arthroplasty devices. Open Rheumatol J.

[17] Van Norman GA (2016). Drugs, devices, and the FDA: Part 2: an overview of approval processes: FDA approval of medical devices. JACC Basic Transl Sci.

[18] Naghshineh N, Brown S, Cederna PS (2014). Demystifying the U.S. Food and Drug Administration: understanding regulatory pathways. Plast Reconstr Surg.

